# Effect of Hyperbaric Oxygen Therapy on Polarization Phenotype of Rat Microglia After Traumatic Brain Injury

**DOI:** 10.3389/fneur.2021.640816

**Published:** 2021-06-03

**Authors:** Fang Liang, Nan Kang, Pinpin Li, Xuehua Liu, Ge Li, Jing Yang

**Affiliations:** ^1^Department of Hyperbaric Oxygen, Beijing Chao-Yang Hospital, Capital Medical University, Beijing, China; ^2^Department of Orthopedics, Beijing Chao-Yang Hospital, Capital Medical University, Beijing, China

**Keywords:** hyperbaric oxygen, traumatic brain injury, microglia, polarization phenotype, neurological function

## Abstract

**Background:** The neurological defect caused by secondary damage following traumatic brain injury (TBI) is considered critical for the management of TBI. Microglia (MG) are a resident brain macrophage that could differentiate into M1 type or M2 type in response to injury and repair. It is known that the MG transition from M1 phenotype to anti-inflammatory M2 phenotype might reduce secondary injury of TBI. So, a TBI animal model was established and we compared biomarkers of M1 and M2MG between the controls and experimental animals receiving hyperbaric oxygen therapy (HBOT). This study aimed to explore whether HBOT was an effective method to improve neural functional recovery *via* promoting the polarization of MG into M2 after TBI.

**Methods:** The rats were randomly divided into four groups: SH (Sham-operated), SH + HBO (hyperbaric oxygen), TBI, and TBI + HBO. Each group included 42 rats, and each of these were divided into the following groups: 1, 6, 12, 24, 72 h, 7, and 14 days. The expression of M1 biomarker inducible nitric oxide synthase (iNOS), M2 biomarker arginase 1 (Arg1), associated cytokine tumor necrosis factor-α (TNF-α), and transforming growth factor-β1 (TGF-β1) was evaluated after the observation time.

**Results:** TBI significantly increased the expression levels of M1 marker iNOS and M2 markers Arg1 at different time points. The increased expression of iNOS was suppressed, while the expression level of Arg1 was enhanced by HBOT. Moreover, HBOT suppressed the pro-inflammatory TNF-α secreted by M1, and promoting the anti-inflammatory TGF-1β.

**Conclusions:** In the present study, HBOT showed the effects on shift of M1 toward M2 phenotype with increased expression of M2 biomarkers and decreased expression of M1 biomarkers in the early stage after TBI.

## Introduction

Traumatic brain injury (TBI) has become a major cause of death and disability in the world mainly due to increasing use of automobiles and development of construction industry (1). Among the surviving TBI patients, 10% of patients with mild injuries would have permanent disabilities, while the 66% of patients would have moderate and severe injuries (2). Primary injuries following TBI include nerve cell and vascular destruction caused by mechanical violence at the moment of accident, while inflammatory responses due to primary damage are the major drivers of secondary injuries. Therefore, reducing inflammatory damage is critical in promoting functional recovery following TBI (3).

As brain-resident macrophage, microglia (MG) provide early immune defense in the central nervous system (CNS). Under normal physiological conditions, the MG are in a resting state during immunological surveillance and defense. Upon cerebral injury, the MG are rapidly activated and polarized to M1 and M2 phenotypes, which have distinct functions in neuroimmunity. The M1 phenotype MG induced by lipopolysaccharide (LPS) or interferon-gamma (IFN-γ) secretes high levels of pro-inflammatory cytokines; the M2 phenotype MG are divided into three subtypes: M2a, M2b, and M2c, which secretes neurotrophic factors and anti-inflammatory cytokines (4).

Hyperbaric oxygen therapy (HBOT) is widely used to promote recovery of the patient from craniocerebral injury, in which the patient inhales pure oxygen or a high concentration of oxygen in an environment that is maintained above one atmosphere (5). Previous studies have demonstrated that HBOT following TBI attenuates microgliosis and proinflammatory cytokine TNF-α expression, resulting in a neuroprotective effect (6, 7). Ming et al. explored the mechanisms of HBO on the regulation of MG polarization *via* its effects on the expression of P-JNK and STAT1. The results suggested that HBOT could reduce M1 polarization and expression of pro-inflammatory cytokines after brain injury, which might be through the JNK/STAT pathway (8).

However, the effect of HBOT on shift of M1 toward M2 phenotype following TBI remained unclear. Hence, in our study, the effects of HBOT on dynamic changes of M1 and M2 microglial markers at different time points following TBI were investigated to explore the potential mechanisms and signal pathway further.

## Materials and Methods

### Experimental Animals

Experimental Sprague–Dawley rats (8 weeks old, weighing 250–500 g, male and female) were provided by the Experimental Animal Center of the Capital Medical University. All animals were housed under a controlled environment at 20°C with food and water *ad libitum*. The rats were fasted for 8 h and deprived of water for 2 h prior to surgery. The present study was approved and performed in accordance with the ethical guidelines of the Committee on the Ethics of Animal Experiments, Capital Medical University (permit no. 2010-D-013; Beijing, China).

### Instruments

The main instruments including HPD-1700 Fluid Percussion Device (DRAGONFLY R&D INC), ASI Small Animal Stereotaxic Instrument (ASI), and DWC150-300 Pure Oxygen Animal Experiment Chamber (Yangyuan Medical Oxygen Cabin Factory of Shanghai 701 Institute) were used.

### Grouping of Experimental Animals

The rats were randomly divided into four groups: Sham-operated (SH), Sham-operated plus hyperbaric oxygen (SH + HBO), traumatic brain injury (TBI), and traumatic brain injury plus hyperbaric oxygen (TBI + HBO).

#### Rat TBI Model

The rats were anesthetized by intraperitoneally injecting 10% chloral hydrate (350 mg/kg) and then were fixed in prone position onto the stereotaxic instrument. Disappearance of corneal reflex and righting reflex was used as indices of successful anesthesia. The rat's head was disinfected and the skin of the right cranial top was cut under sterile conditions to expose the skull. A 3-mm-diameter hole was drilled through the right parietal bone (4 mm from the skull herring bone and 3 mm lateral from the sagittal suture), leaving the dura mater intact. A 3-mm-diameter percussion tube was adhered to the drill hole with cyanoacrylate adhesive glue and zinc phosphate cement, followed by suturing of the surrounding skin. The reservoir tube of the hydraulic percussion instrument was filled with physiological saline at 37°C, and the angle and weight of the percussion hammer were preset. The reservoir tube was connected to the percussion tube, and the hammer was released to let the saline solution impact the cortical surface. The signs of successful percussion included edema and hemorrhage as evidenced by purple-red discoloration and bulging of dura mater. After percussion injury, the percussion tube was removed, the skull hole was closed with bone wax, and the incision was completely sutured.

#### Sham Surgery

Anesthesia, disinfection, skin incision, and skull drilling were performed as described in the above TBI model section, except that the skull hole was closed with bone wax without percussion injury prior to suturing.

### Hyperbaric Oxygen Therapy

The chamber was perfused with pure oxygen for 5 min and then the pressure was increased to 2 ATA for over 10 min. The animals inhaled HBO for 60 min at stable pressure and then decompressed for 15 min. During HBO treatment, the chamber was continuously ventilated for 8–10 L/min to prevent CO_2_ accumulation and maintain the oxygen concentration at 95%. The chamber temperature was maintained at 22–26°C, and the humidity was between 40 and 50%. The rats in the non-HBO groups were placed in the chamber for the same duration but inhaled oxygen at atmospheric pressure. In both groups, HBOT was given daily at 11 a.m. for 14 consecutive days.

### Evaluation of Neural Function and Sample Collection

The recovery of neural function was evaluated by the modified neurological severity (mNSS) scores at 1, 6, 12, 24, 72 h, 7, and 14 days after surgery (9). The mNSS score test includes assessment of motor, sensory, balance, and reflex functions. The tests were conducted by observers who were blinded to the experimental conditions and treatments. The mNSS score was graded on a scale of 0 to 14, where normal score was 0, and maximal deficit score was 14. One score point represents the inability to perform the test or lack of a tested reflex; 10–14: severe; 5–9: moderate; and 1–4: mild injuries.

Following the evaluation of neural function, the animals were sacrificed using chloral hydrate. The sternum was cut to expose the heart, and the left ventricle was injected with 0.2 ml of 1% heparin sodium. The aortic vessel via the left ventricle and the auricula dextra were cut. The blood vessels were then quickly perfused with 150 ml of normal saline. After that, the rats were decapitated to obtain the brain tissue from the cranium without traumatization. The brain tissue in the periphery of the injury site was carefully extracted and divided into two parts. One part was stored in 4% paraformaldehyde for performing immunohistochemistry and terminal deoxynucleotidyl transferase-mediated dUTP nick end labeling (TUNEL) assay, and the other stored was in a refrigerator at −80°C for PCR and Western blot analysis.

### Laboratory Tests

#### Reverse Transcription Quantitative Polymerase Chain Reaction

Total RNA was extracted from the frozen brain tissue by routine methods and quality was tested by 1% agarose gel electrophoresis. Reverse transcription was performed using the TIANScript RT Kit. A 12- to 18-μl reaction mixture containing 3 μl of template RNA and 0.5 μl of primer (50 μM) was prepared, heated at 70°C for 5 min for denaturation, and then quickly cooled on ice for over 2 min. The RNA/primer denatured solution was precipitated by centrifugating for several seconds. The RT reaction system included template RNA/primer pellet, 5 μl of 5 × M-MLV Buffer, 1.25 μl of dNTP mixture (10 mm of each nucleotide), 25 U of RNase inhibitor (40 U/μl), 200 U of M-MLV (200 U/μl), and RNase-free dH_2_O in a total volume of 25 μl. The RT reaction was run at 42°C for 2 h and 95°C for 15 min. Real-time PCR was performed using a SYBR FAST qPCR Kit (KAPA Biosystems, USA). The amplified iNOS and Arg1 were 283 and 216 bp, respectively (Table 1). The 2^−Δ*ΔCT*^ method was used to calculate the relative expression levels of target mRNAs. GAPDH was used as an internal control.

**Table 1:** Primers for qRT-PCR.

**Primer sequences**	**Primer sequences**	**Probes**	**Sizes of PCR products**
iNOS	Forward primer	5′-TAGTCAACTACAAGCCCCACG-3′	283 bp
iNOS	Reverse primer	5′-AGTCACATGCAGCTTGTCCA-3′	
Arg1	Forward primer	5′-GGACATCGTGTACATCGGCT-3′	216 bp
Arg1	Reverse primer	5′-GTAGCCGGGGTGAATACTGG-3′	

#### Western Blotting

Total protein was extracted from 20 μg of frozen tissue in 400 μl of protein lysis buffer by homogenization on ice. The homogenates were then transferred to pre-chilled 1.5-ml EP tubes, placed on ice for 15 min for complete lysis, and centrifuged at 12,000 rpm for 10 min at 4°C. The supernatants were transferred to 0.5-ml centrifuge tubes and stored at −20°C. A 50- to 100-μg sample was mixed with 5 × loading buffer, heated in boiling water for 5 min, quickly cooled, and loaded onto a polyacrylamide stacking gel for electrophoresis. The stacking gel was run at 80 V and separation gel was run at 120 V. The separated proteins were then transferred to the polyvinylidene difluoride (PVDF) membranes. The membranes were then washed with tris-buffered saline and 1% Triton (TBS-T) for 5 min, followed by incubation with the primary antibody diluted in blocking solution at 4°C overnight. The membranes were washed three times with TBS-T (5 min per wash) and then incubated for 2–3 h with a horseradish peroxidase (HRP)-labeled secondary antibody (Abcam, Lot No. Ab60176; Abcam, Lot No. Ab15323) diluted in blocking buffer. The blotted membranes were washed again with TBS-T (three times, 5 min each), and the bands were visualized with ECL chemiluminescence reagent. The gray-scale images were analyzed using imaging software.

#### Immunohistochemistry

The tissue samples were fixed in formalin, and the inducible nitric oxide synthase (iNOS) and arginase-1 (Arg1) were investigated by immunohistochemical analysis. Liver arginase antibody (goat 1:50) (Abcam, Lot No. Ab60176) and iNOS antibody (Rabbit 1:100) (Abcam, Lot No. Ab15323) were purchased from Abcam company (Cambridge, UK).

The paraffin-embedded tissue samples were sectioned, placed in EDTA antigen retrieval solution, and heated by microwave. After cooling, the sections were washed three times (5 min/wash) with phosphate buffered saline (PBS, pH 7.4) by shaking, incubated in 3% hydrogen peroxide solution for 25 min at room temperature in a dark environment to quench the endogenous peroxidase activity, washed with PBS (three times for 5 min each), drip-coated with 3% BSA for 3 min, and then incubated with pre-diluted primary antibody solution at 4°C overnight. After washing in PBS, the sections were incubated with HRP-labeled secondary antibodies at room temperature for 50 min. After washing and drying, DAB solution was added for staining. Color development time was controlled by microscopic observation. For qualitative evaluation, the brown-yellow staining was considered positive. After color development was terminated by running water, the sections were counterstained with hematoxylin, dehydrated, and then mounted. The presence of brownish yellow granules was considered as positive protein expression. The average optical density (AOD) of positive staining in five fields per section and five sections per rat was analyzed using Image-Pro Plus 6.0 (Media Cybernetics, USA) software. All samples were analyzed by two double-blinded pathologists under high-times optical microscope.

#### Enzyme-Linked Immunosorbent Assay

The levels of pro-inflammatory TNF-α and anti-inflammatory TGF-1 in the tissue homogenates were measured using ELISA kits (Abcam, UK).

The main instruments used for ELISA were a Multiskan Mk3 Microplate Reader (Thermo, USA), a DH4000A electric thermostat incubator (Tianjing Taisite, China), and an MH-1 mini shaker (Kylin–Bell Lab Instruments, China). The main reagents included rat transforming growth factor (TGF)-β1 antibodies (Abcam, Lot No. ab119558 GR3192499-2) and rat tumor necrosis factor (TNF)-α antibodies (Abcam, Lot No. ab46070 GR3191907-6).

Standards were prepared at five dilution gradients and 50 μl of each transferred to individual microplate wells. The blank wells and sample wells were then prepared accordingly. Briefly, 40 μl of dilution solution was mixed with 10 μl of sample solution by gentle shaking. The plate was covered with sealing film and incubated at 37°C for 30 min. The solution in the wells was discarded and diluted washing solution was then added to each well for 30 s. After five 30-s washes, 50 μl of diluted detection antibodies was added to each well (except blank control wells) and the plate was covered with sealing film. After 30 min of incubation at 37°C, the wells were washed as described above, followed by sequential addition of 50 μl of color developing solutions A and B through mixing. The plate was then incubated at 37°C for 15 min, and then 50 μl of stop solution was added. After 15 min, the optical density (OD) was measured at 450 nm. The OD of the blank wells was subtracted from the OD of the sample wells. The OD values of the standards were fitted by a polynomial quadratic regression equation to generate standard curves for quantifying the target proteins using an appropriate dilution factor.

#### TUNEL Method

The tissue samples that were fixed in formalin were embedded in paraffin. Four-millimeter-thick sections obtained from the paraffin blocks were affixed to positively charged slides. The apoptotic cells in the sections were shown with TUNEL assay by using Apoptosis Kit (11684817910, Roche, Basel, Switzerland). The sections were maintained at 37°C for one night. The sections that were deparaffinized by passing through xylol and reduced alcohol sequences were washed in the PBS (Zhongke Wanbang Biotechnology, Beijing, China). The sections were then incubated in an incubator with proteinase K (Zhongke Wanbang Biotechnology, Beijing, China) enzyme for 15 min at 37°C. Next, 3% H_2_O_2_ (7722-84-1, Sinopharm Chemical Reagent Co., Ltd, Shanghai, China) prepared in the PBS was applied to the sections in a dark environment for 5 min to prevent endogenous peroxidase activity. TdT enzyme was applied to the sections for 1.5 h and then kept in the balancing buffer in an incubator for 10 min at 37°C. To stop the reaction of TdT enzyme, after applying the stopping/washing buffer in the kit for 20 min at room temperature, the sections were washed three times in the PBS. The anti-digoxigenin peroxidase enzyme was then applied in an incubator at 37°C. After washing in the PBS, 3.3′-diaminobenzidine (DAB) substrate was applied to the sections as chromogen. After application for 30–45 min, the reaction in brown color was stopped by using the distilled water. After that, the sections were stained with methyl green, which were used as the opposite dye, and rapidly passed through butanol. The slides that were made transparent by applying xylol for 15 min in total were covered with neutral gum and prepared for review using a light microscope (NIKON CI-S). The apoptotic index was determined independently by two blind observers by counting under 200 × magnification and randomly selected 8–10 different fields containing for all individuals groups. The apoptotic Index was calculated using the formula 100% × (TUNEL-number of positive cell nucleus/number of total cell nucleus).

### Statistical Analysis

Statistical analysis was performed using SPSS23.0 software. All variables were initially tested for normality. The measurement data are expressed as means ± standard deviation. The neurological function scores (mNSS) were compared by the Student's *t*-test, and comparisons among multiple groups were performed using one-way analysis of variation (ANOVA). *P* < 0.05 (two-tailed) was considered to be statistically significant for all tests.

## Results

### HBOT Improved Neural Functional Recovery Following TBI

**Figure 1:**
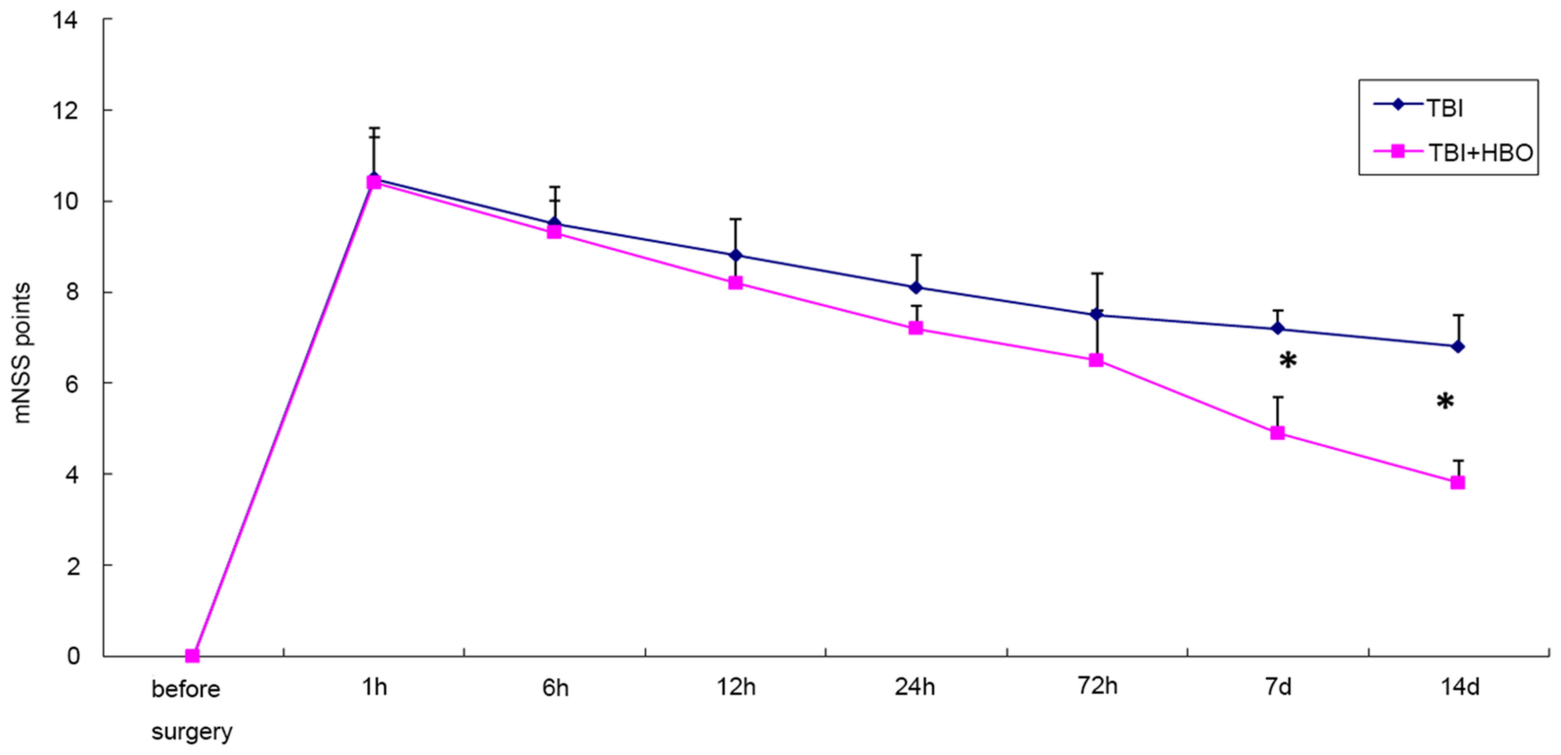
mNSS scores in TBI and TBI + HBO groups. Data are presented as means ± standard deviation. *P < 0.05, TBI + HBO group vs. TBI group.

The NSS scores of the SH and SH + HBO groups were zero. The NSS scores were significantly increased after surgery in the rats of TBI groups. The NSS scores were significantly reduced in the 7- and 14-day TBI + HBO groups, compared to the TBI groups (*P* < 0.05). This suggested that HBOT could significantly improve neural functional recovery following TBI (Figure 1).

### HBOT Inhibited TBI-Induced Increase of M1 Marker iNOS

After TBI, the MG polarized toward M1 phenotype and expressed high levels of iNOS, inducing nitric oxide production. An extensive transcription profile was examined in our experiment.

**Figure 2:**
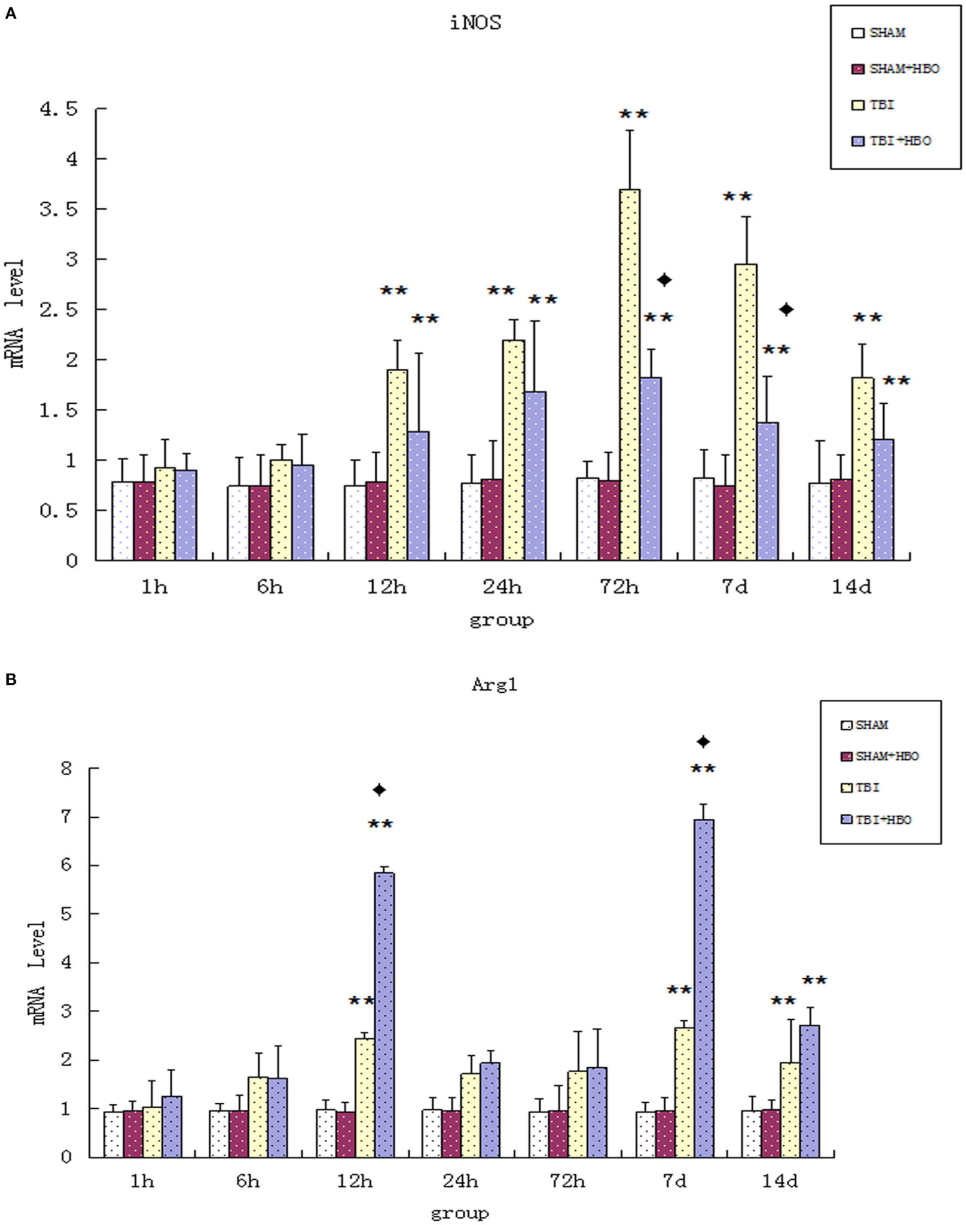
**(A)** RT-qPCR analysis of mRNA levels of iNOS in the brain tissues of all rat groups at different time points following surgery. **(B)** RT-qPCR analysis of mRNA levels of Arg1 in the brain tissue of all rat groups at different time points following surgery. Data are presented as means ± standard deviation. **P < 0.05. TBI and TBI + HBO groups vs. SH and SH + HBO groups; ^♦^*P* < 0.05, TBI + HBO group vs. TBI group.

**Figure 3:**
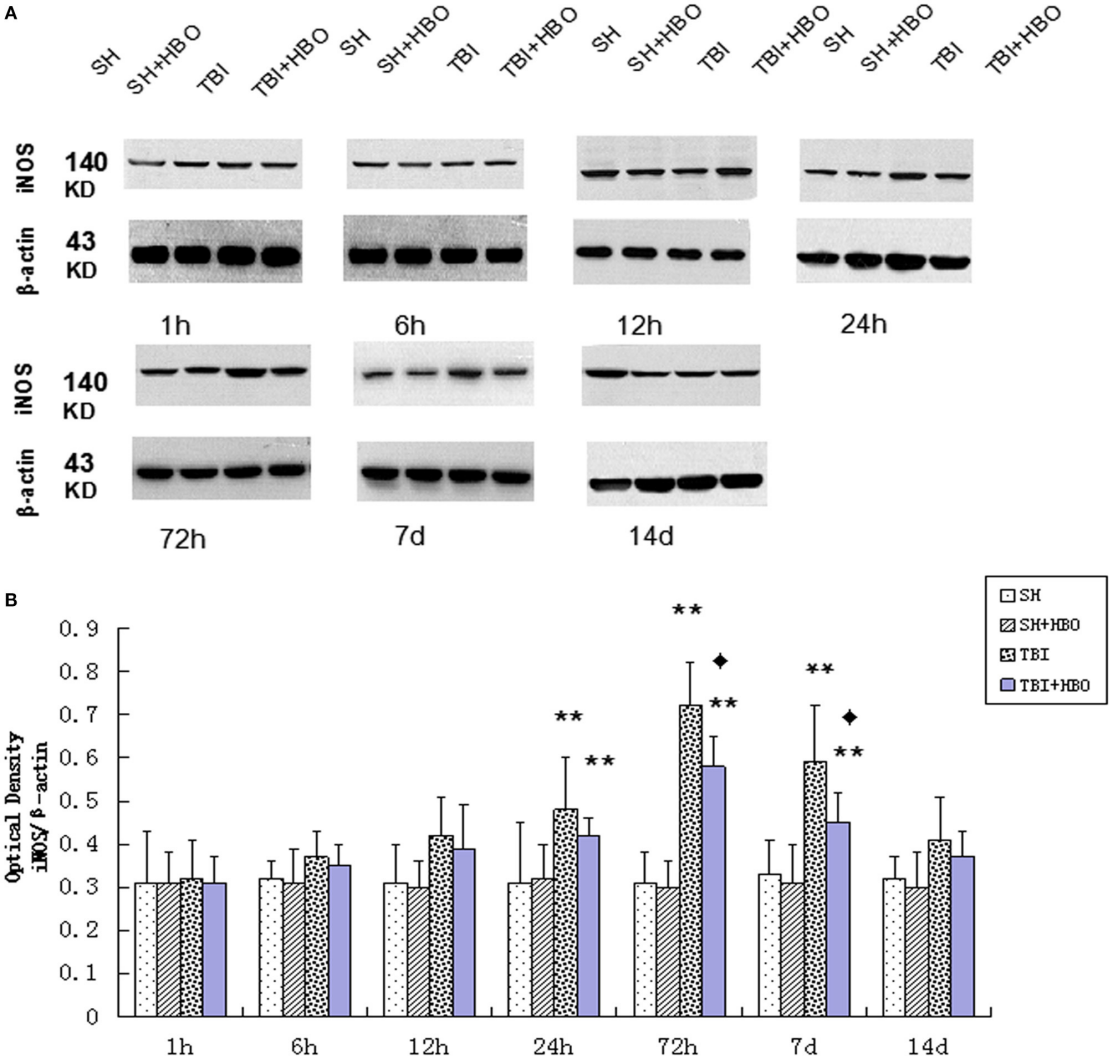
**(A)** Western blotting of iNOS in all groups. **(B)** Quantification analysis of iNOS. Data are presented as means ± standard deviation. **P < 0.05, TBI and TBI + HBO groups vs. SH and SH + HBO groups; ^♦^*P* < 0.05, TBI + HBO group vs. TBI group. The loading control images have been re-used in both Figures 3, **5** for illustrative purposes.

Firstly, the mRNA and protein levels of M1 microglial marker iNOS were detected by RT-q PCR and Western blotting. Both the gene (Figure 2A) and protein (Figure 3) expressions were gradually increased in the TBI groups, peaking at 72 h. However, the expression levels were lowered in the TBI + HBO groups, compared to the TBI groups. The difference was statistically significant at 72 h and 7 days post-TBI (*P* < 0.05).

**Figure 4:**
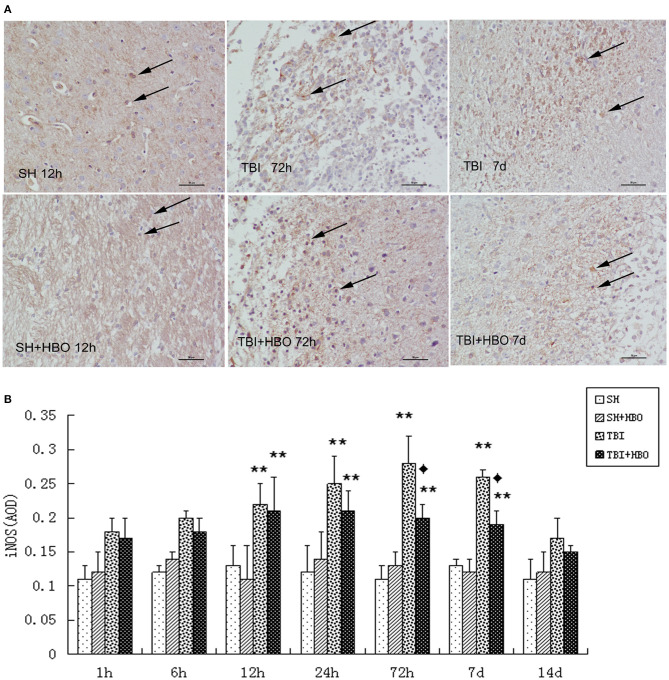
**(A)** Immunohistochemical staining of iNOS in the brain tissue samples, and the brown staining was considered positive cells. Bar: 50 μm. **(B)** Analysis of the average optical density of iNOS in each group. Data are presented as means ± standard deviation. **P < 0.05, TBI and TBI + HBO groups vs. SH; ^♦^*P* < 0.05, TBI + HBO group vs. TBI group.

To further confirm the outcome of induction of polarizing M1 by TBI, immunohistochemistry staining of iNOS was performed at the site of injury. In accordance with mRNA and protein expression measurements, The AOD of iNOS was significantly lowered in the TBI + HBO groups, compared to the 72-h and 7-day TBI groups (*P* < 0.05), as shown in Figure 4.

These data indicate that, as the M1 microglial marker, iNOS were rapidly increased in the early stage of TBI and diminished by HBOT administration effectively.

### HBOT Enhanced the Level of M2 Markers Arg1 Following TBI

**Figure 5:**
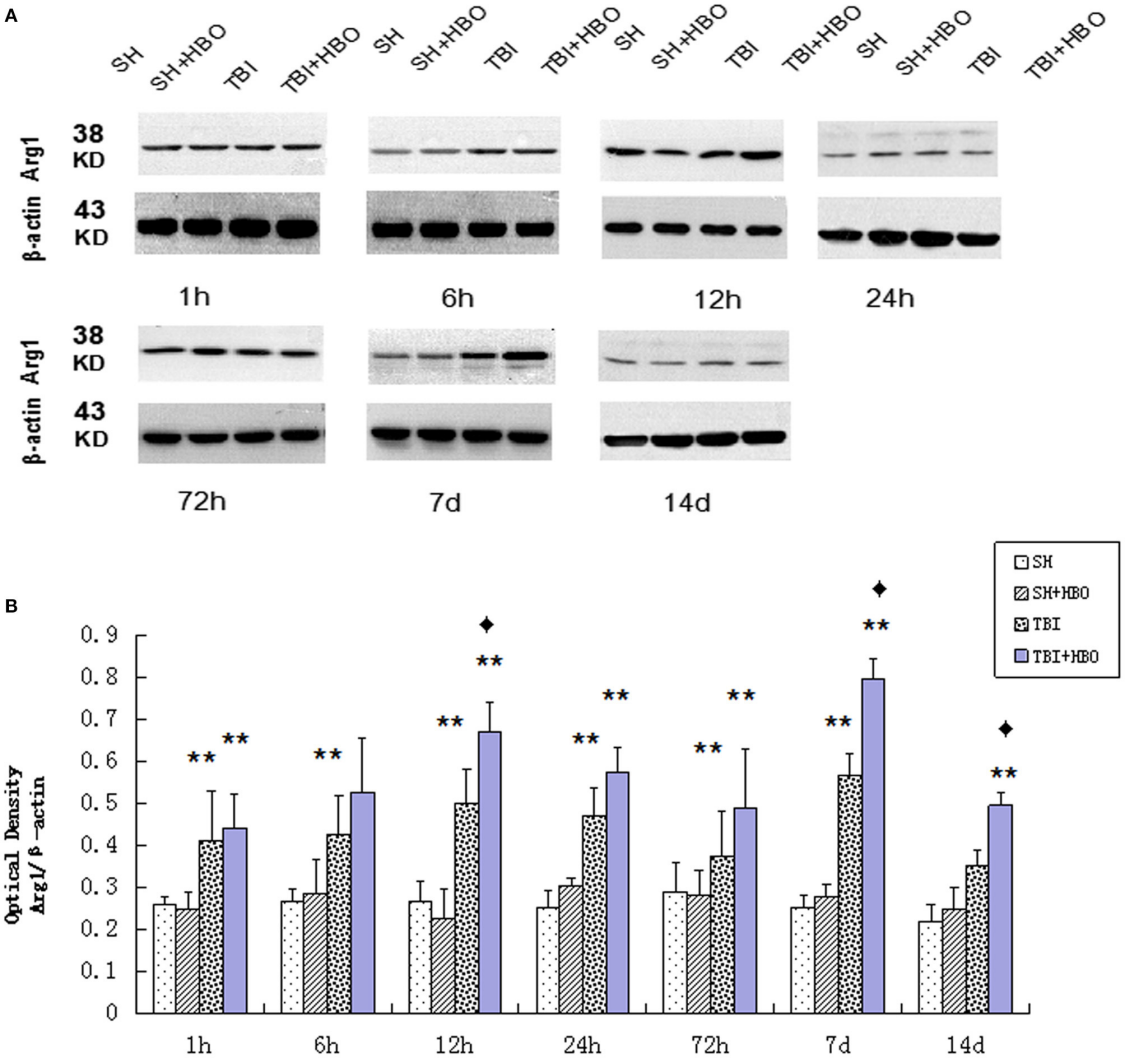
**(A)** Western blotting of Arg1 in all groups. **(B)** Quantification analysis of Arg1. Data are presented as means ± standard deviation. **P < 0.05, TBI and TBI + HBO groups vs. SH and SH + HBO groups; ^♦^*P* < 0.05, TBI + HBO group vs. TBI group. The loading control images have been re-used in both Figures 3 and 5 for illustrative purposes.

Arg1 mRNA (Figure 2B) and protein (Figure 5) expressions detected displayed an increase after TBI, eventually reaching peak within 7 days in TBI groups. As the M2 markers, the Arg1 expression levels were enhanced significantly by HBO at 12 h and 7 days, lasting 14 days (*P* < 0.05).

**Figure 6:**
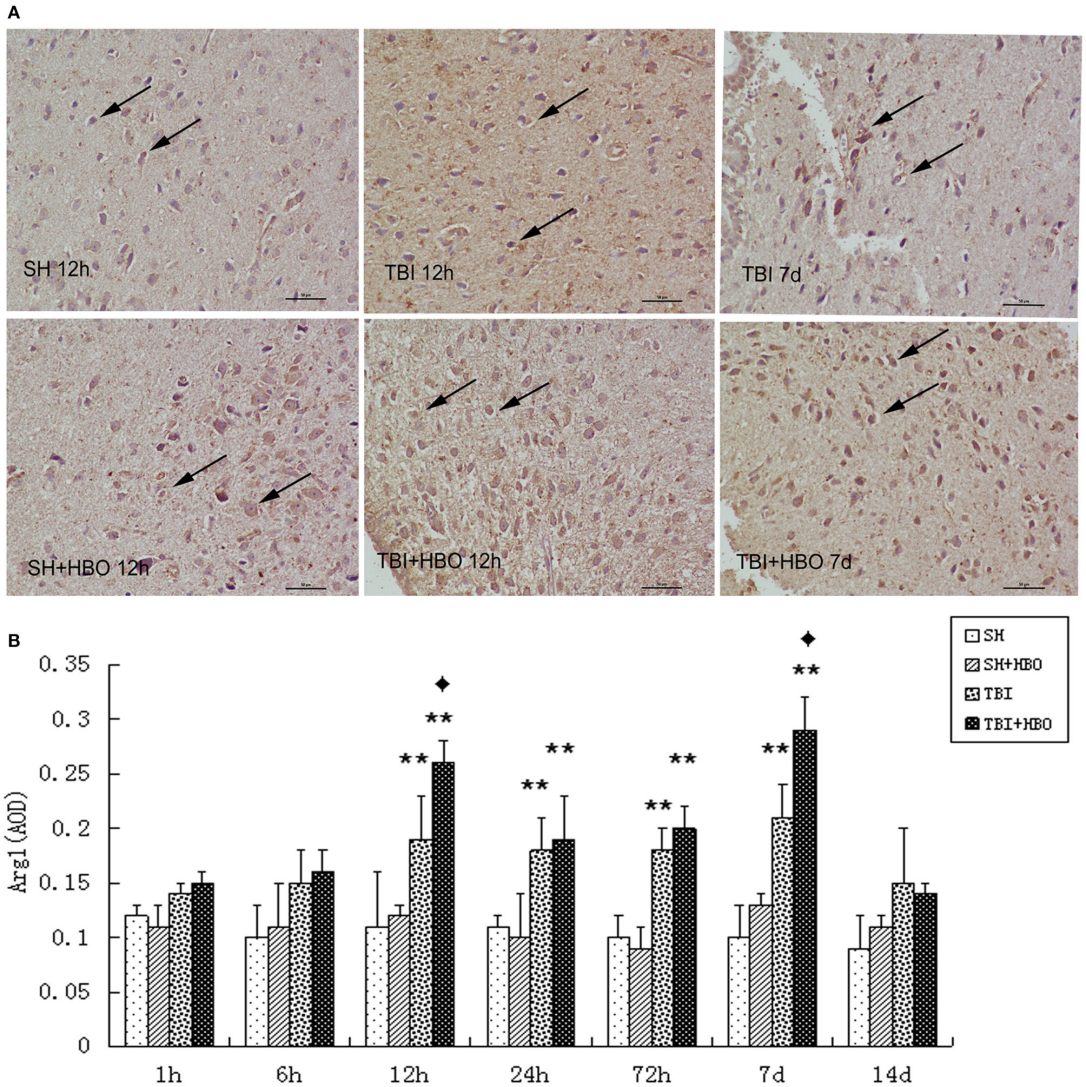
**(A)** Immunohistochemical staining images of Arg1 in the brain tissue samples, in which the brown staining was considered positive cells. Bar: 50 μm. **(B)** Analysis of the average optical density of Arg1 in each group. Data are presented as means ± standard deviation. **P < 0.05, TBI and TBI + HBO groups vs. SH; ^♦^*P* < 0.05, TBI + HBO group vs. TBI group.

In the same way, immunohistochemistry staining showed the presence of significantly more Arg1-positive MG at the center of the injury after TBI, and this was in accordance with the RT-qPCR and Western blotting results. The AOD of Arg1 was significantly higher in the TBI + HBO groups than that in the 12-h and 7-day TBI groups (*P* < 0.05), as shown in Figure 6.

In contrast with M1, the expression of M2 markers (Arg1) was significantly enhanced by HBOT (12 h, 7, and 14 days). Taken together, these results demonstrated that HBOT could significantly alter the M1/M2 phenotype ratio, via inhibiting M1 and enhancing M2 expression after TBI.

### HBOT Down-Regulated the Release of TNF-α Secreted by M1 MG

The results of ELISA showed that the levels of pro-inflammatory cytokine TNF-α secreted by M1 MG significantly increased after TBI. The levels reached peak at 24 h (1291.30 ± 19.81 pg/ml) in the TBI group. Compared with TBI groups, the TNF-α levels in the TBI + HBO group were significantly decreased at 24 h and 72 h after surgery (*P* < 0.05, Table 2).

**Table 2:** Levels of TNF-α in brain tissue following SH and TBI with or without HBO (in pg/ml).

**Group**	**SH**	**SH + HBO**	**TBI**	**TBI + HBO**
1 h	704.06 ± 22.89	699.47 ± 12.06	768.36 ± 13.75^**^	763.36 ± 21.22^**^
6 h	702.98 ± 11.58	695.55 ± 21.83	834.49 ± 21.99^**^	821.51 ± 10.73^**^
12 h	692.31 ± 20.66	700.74 ± 15.66	1325.30 ± 32.61^**^	1281.02 ± 31.00^**^
24 h	702.42 ± 20.18	697.21 ± 12.33	1291.30 ± 19.81^**^	1055.46 ± 27.75^**^^♦^
72 h	705.17 ± 11.88	702.69 ± 21.44	960.83 ± 25.75^**^	882.52 ± 24.36^**^^♦^
7 d	698.02 ± 13.75	696.71 ± 17.98	847.76 ± 23.77^**^	814.24 ± 14.98^**^
14 d	706.24 ± 20.86	697.44 ± 20.26	795.42 ± 30.57^**^	781.89 ± 23.19^**^

*Data are presented as the mean ± standard deviation.*

** *P < 0.05, TBI and TBI + HBO groups vs. SH and SH + HBO groups*

♦ *P < 0.05, TBI + HBO group vs. TBI group*.

Interestingly, accompanied with elevation of iNOS, TNF-α displayed the prominent increase at 24 h following TBI, which was down-regulated by HBO. These results suggested that HBOT alleviated TBI-induced M1 microglial activation, thus inhibiting the subsequent inflammatory cytokine.

### HBOT Up-Regulated the Release of M2 Associated Cytokine TGF-1β Following TBI

According to the previous studies, the apparent increase in M2 phenotype expression might be associated with the reduction of NO production and up-regulation in anti-inflammatory cytokines TGF-1β. So, in our experiment, TGF-1β was examined by ELISA. The results showed that the TGF-1β levels were significantly higher in the TBI + HBO groups than that in the TBI groups at 72 h and 7 days (*P* < 0.05, Table 3).

**Table 3:** Levels of TGF-1β in brain tissue following SH and TBI with or without HBO (in pg/ml).

**Group**	**SH**	**SH + HBO**	**TBI**	**TBI + HBO**
1 h	1234.8 ± 3.59	1238.6 ± 4.5	1275.5 ± 3.6^**^	1214.6 ± 3.6^**^
6 h	1198.9 ± 3.8	1271.8 ± 3.1	2015.0 ± 9.3^**^	2157.2 ± 8.8^**^
12 h	1213.92 ± 3.8	1207.0 ± 3.4	2467.2 ± 3.5^**^	2800.90 ± 7.6^**^
24 h	1228.4 ± 7.2	1238.3 ± 3.9	2344.5 ± 11.0^**^	4265.3 ± 18.5^**^^♦^
72 h	1286.3 ± 27.6	1271.6 ± 7.1	1948.4 ± 15.5^**^	2614.9 ± 15.4^**^>^♦^
7 d	1242.7 ± 6.3	1198.7 ± 6.2	1689.5 ± 5.2^**^	2247.9 ± 10.1^**^>^♦^
14 d	1236.9 ± 4.1	1215.4 ± 4.5	1563.3 ± 6.0^**^	1626.4 ± 9.7^**^

*Data are presented as the mean ± standard deviation.*

** *P < 0.05, TBI and TBI + HBO groups vs. SH and SH + HBO groups*

♦ *P < 0.05, TBI + HBO group vs. TBI group*.

The up-regulation of TGF-1β by HBO manifested transformation of nervous tissue from a proinflammatory to an anti-inflammatory state after TBI and improved the immune microenvironment to allow neuronal survival.

### HBOT Attenuated TBI-Induced Apoptosis by Modulating MG Polarization

**Figure 7:**
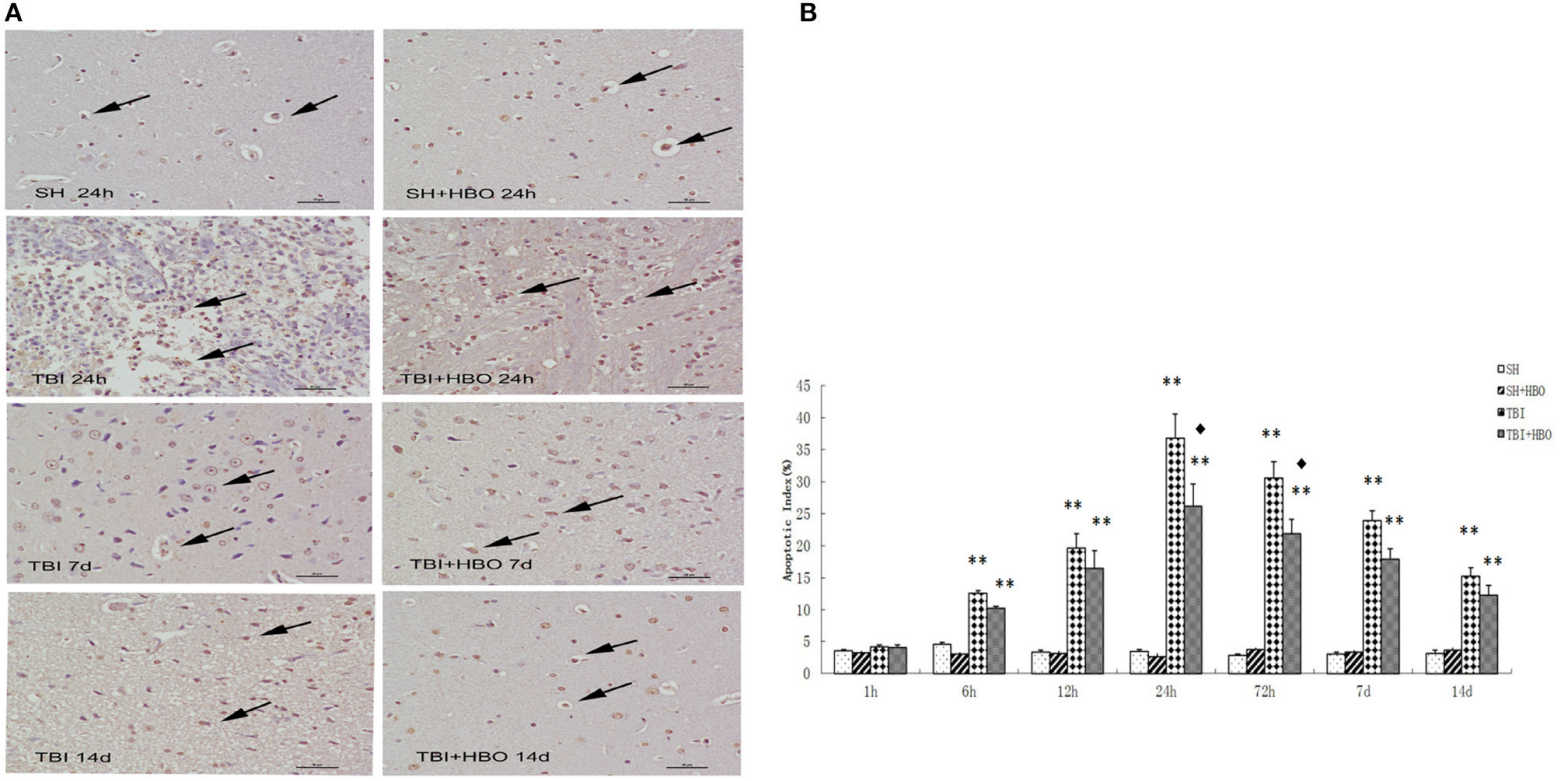
**(A)** TUNEL staining of Arg1 in brain tissue samples, and brown staining was considered positive cells. Bar: 50 μm. **(B)** Apoptotic index (%) in each group. Data are presented as means ± standard deviation. **P < 0.05, TBI and TBI + HBO groups vs. SH and SH + HBO groups; ^♦^*P* < 0.05, TBI + HBO group vs. TBI group.

To further confirm the anti-apoptosis effect of HBOT via regulation of MG polarization following TBI, TUNEL staining was used to detect neuronal apoptosis in the sections of injured brain tissue. The TUNEL-positive cells were increased at the sites of injury in TBI groups when compared to SH and SH + HBO groups, reaching peaks at 24 h in the TBI group. However, the apoptotic index was significantly lower in the TBI + HBO groups, compared to the TBI groups at 24 and 72 h (*P* < 0.05, Figure 7).

## Discussion

It is well-known that the MG are generally in a resting state. M1 and M2 MG synergistically regulate inflammation, remove debris, and promote tissue remodeling and repair. However, homeostasis was disturbed after TBI. Activation of MG transforms from multi-branched to amoeba-like cells, exhibiting a myriad of new biological properties, including proliferation, chemotaxis, cytophagy, and migration (10, 11). Majority of MG at the site of TBI have mixed M1- and M2-type activation profiles, secreting different cytokines (12, 13).

In this study, qRT-PCR and Western blotting were used to estimate the mRNA and protein expression levels of M1-type specific marker iNOS in the rat injured brain tissues following experimental TBI. After 12 h of TBI, the expression of iNOS was increased, reaching peak on day 3 post-TBI. In accordance with RT-qPCR, immunohistochemical staining results revealed a significant increase in AOD of iNOS in TBI groups. A previous study confirmed that reactive oxygen species (ROS) played key inflammatory mediators after TBI, contributing to cytokine release and microglial polarization. Farag et al. (14) found NADPH-oxidase (Nox) activation after TBI, which could cause ROS generation and the MG prone to M1 polarization. It provided an explanation for our result that iNOS was increased following TBI but expression levels were unchanged in SH and SH + HBO groups. In addition, ELISA results revealed that cytokine TNF-α secreted by M1 MG reached peak on day 1 post-TBI. As the initiation factors of cytokine network, TNF-α might induce the production of other inflammatory factors to launch an inflammatory cascade response further (15–17). Different from M1, the M2 biomarker Arg1 was increased later and M2 polarization reached peak in Arg1 on day 7 after injury. Our results are roughly consistent with that of the previous work, which documented M2 numbers reaching peak after 5 days of stroke (18, 19). It is inferred that the M1-type MG were predominated during the early stage of secondary injury, aggravating necrosis and apoptosis of neurons (20, 21). So, effective therapeutic targets for TBI would be focused on changing the MG polarization states. We speculated that the effect of HBOT on TBI might be transition of MG from M1- to M2-dominant phenotypes.

How did HBOT alter the M1/M2 ratio to improve the functional recovery in the early stage of TBI? In our experiment, the expression of iNOS was substantially reduced by HBOT, strongly suggesting that HBOT might effectively suppress M1 MG polarization and pro-inflammatory cytokines activation after TBI. We performed 2 ATA HBOT in the present study. The effects of HBOT were increased solubility and diffusion of gas (O_2_) which could improve hypoxia state of damage tissue. Appropriate O_2_ supplementation could attenuate the TBI-induced ROS accumulation. Previous studies have reported that HBOT has been shown to reduce secondary damage following TBI by improving oxidative metabolism, reducing free radicals mediated damage and neuro-inflammatory response (22–24). Lin et al. (25) reported observing attenuated inflammation in rats with TBI following 3 days of 2 ×1 h 2.0 ATA HBOT, evidenced by decreased brain myeloperoxidase activity. So, we hypothesized that HBOT inhibited the p38a MAPK signaling pathway by mitigating oxidative stress, which was considered critical for the activation of M1 subtype. Meanwhile, the gene and protein expression of M2 marker Arg1 were found to be higher by HBOT at 12 h after TBI, confirming a shift from M1- to M2-type MG in the early stage of injury. By further exploration, the increased Arg1 by HBOT was speculated to competitively bind to L-arginine, which is the common substrate of iNOS, thereby reducing iNOS production by M1 MG (26–28). In addition, as anti-inflammatory cytokines secreted by M2 MG, TGF-β1 was further increased by HBO accompanied with Arg1. Interestingly, increased TGF-1 expression and decreased TNF-α expression were detected simultaneously in the TBI + HBOT groups. These data supported the mechanism that TGF-1 might be associated with a reduction in TNF-α production (29). Increased TGF-β1 by HBO might regulate autoimmune responses through an autocrine mechanism, thereby inhibiting the release of TNF-α by M1 (30).

From histologic analysis, the apoptotic index was significantly lower in the TBI + HBO group, compared to the TBI group at 24 h. It was consistent with the expression change of TNF-α by HBO. Possibly, HBOT could effectively reduce apoptotic cell by down-regulating the protein expression of TNF-α secreted by M1 MG, which was the major extrinsic inducer of p53-dependent mitochondrial apoptotic pathway (31, 32). Thus, the effect of HBOT on inhibiting apoptosis might be partly due to its modulation of MG polarization.

The M1 and M2 polarization pathways are influenced by multiple factors including the duration, location, and severity of injury (33–35). HBOT could improve the state of ischemia and hypoxia in the site of TBI. Thus, our study speculated that HBOT might provide a micro-environment conducive to promote the polarization of MG to M2 phenotype.

There are several limitations in this study: (a) the effects of 2 ATA HBOT were only tested during acute phase after TBI. The effect of different ATA HBOT on MG polarization should be tested. (b) In-depth study need to be carried out, such as *in vitro* experiments.

## Conclusion

We conclude that HBOT might alleviate secondary injury and promote nerve repair by promoting M1 MG polarization to M2 following TBI. The study provided a new paradigm of HBOT in TBI.

## Data Availability Statement

The original contributions presented in the study are included in the article/supplementary material, further inquiries can be directed to the corresponding author/s.

## Ethics Statement

The present study was approved and performed in accordance with the ethical guidelines of the Committee on the Ethics of Animal Experiments, Capital Medical University.

## Author Contributions

FL and JY: study design, data collection and analysis, statistical analysis, and manuscript drafting. NK: study design and data collection and analysis. PL: study design and data collection. XL: data collection. GL: manuscript revision. All authors contributed to the article and approved the submitted version.

## Conflict of Interest

The authors declare that the research was conducted in the absence of any commercial or financial relationships that could be construed as a potential conflict of interest.
